# Analysis of high-density single-nucleotide polymorphism data: three novel methods that control for linkage disequilibrium between markers in a linkage analysis

**DOI:** 10.1186/1753-6561-1-s1-s160

**Published:** 2007-12-18

**Authors:** Kristina Allen-Brady, Benjamin D Horne, Alka Malhotra, Craig Teerlink, Nicola J Camp, Alun Thomas

**Affiliations:** 1 Department of Biomedical Informatics, University of Utah, 391 Chipeta Way, Suite D, Salt Lake City, Utah 84108, USA; 2 Genetic Basis of Human Disease Division, Translational Genomics Research Institute, 445 North 5th Street, Phoenix, Arizona 85004, USA

## Abstract

We performed a multipoint linkage analysis for rheumatoid arthritis (RA) using high-density single-nucleotide polymorphism (SNP) data for chromosome 6 and chromosome 21 using Genetic Analysis Workshop 15 (GAW15) data. These regions were previously shown to have high LOD scores, not accounting for linkage disequilibrium (LD). We propose three novel methods to control for LD in a linkage analysis: allow for LD between markers using graphical modeling, eliminate high-LD markers by principal-component analysis (PCA) using haplotype data, and eliminate high-LD markers by PCA using genotype data. All three novel methods were compared to the previously published SNPLINK high-LD elimination method. Although all four methods verified the previous results, differences in linkage peak height and position were observed across methods. Additional work is required to further understand the effects of LD on linkage results and explore LD control methodology.

## Background

The recent availability of rapid, accurate, and relatively low-cost genome-wide, high-density single-nucleotide polymorphism (SNP) panels is changing the study of many complex diseases. Linkage analysis to track inheritance of chromosomal regions in pedigrees no longer must rely on highly informative but sparsely spaced conventional microsatellite markers. Rather, SNPs, which are far more abundant than microsatellite markers, have the capacity to yield a higher information content, and hence an improved potential for localizing disease genes [1].

Classical linkage analysis assumes linkage equilibrium between markers. Applying classical linkage packages to genome-wide SNP panels may generate biased results, if parental genotypes are missing, as SNP panels will include markers that are in linkage disequilibrium (LD). LD between markers may inappropriately weight shared haplotypes in the likelihood calculations, which can lead to inflated LOD scores, resulting in false-positive evidence of linkage [2]. To overcome the potential bias in a linkage analysis by using SNP data, ideally LD must either be incorporated into the analysis or markers in high LD must be eliminated. The commonly used, freely available SNPLINK [3] program eliminates high-LD SNPs using a simple algorithm between contiguous pairs of SNPs. However, we have previously shown that a non-contiguous LD structure is more likely to model the complex pattern of recombination and mutation that have been observed to exist in candidate genes [4,5].

Here we propose three novel methods to control for LD between markers in a linkage analysis that do not require a contiguous LD structure: one method that allows for LD between markers using graphical modeling and two methods that eliminate markers in high LD using principal components. Our three novel methods were compared to the previously published analysis generated by the SNPLINK [3] program. We used genome-wide scan data made available for the Genetic Analysis Workshop (GAW15), Problem 2. The North American Rheumatoid Arthritis Consortium (NARAC) provided 5744 SNPs from a genome-wide scan of 757 families, approximately 90% of which were Caucasian. The NARAC Caucasian families were previously analyzed by Amos et al. [6], who found their two highest linkage results on chromosome 6 (LOD = 18.53) and chromosome 21 (LOD = 11.59), not accounting for LD. However, when markers in high LD were eliminated using SNPLINK [3], Amos et al. [6] found that the chromosome 6 peak remained (LOD = 16.14) but the chromosome 21 peak disappeared (LOD = 1.11). By reanalyzing the chromosome 6 and 21 peaks with three novel LD control methods, we verify the previous results and suggest alternative methods for controlling for high LD without requiring contiguous LD structure.

## Methods

Details regarding NARAC subject enrollment, phenotype designation, and genotype information are published elsewhere [6]. Using the total 404 SNPs available for chromosome 6 and the 104 SNPs available from chromosome 21, we checked for genotype errors using the software CheckErrors [7], which uses graphical modeling to calculate the posterior probability of genotype errors in pedigrees. For chromosome 6 we eliminated 16 SNPs and for chromosome 21 we eliminated 1 SNP because of errors. We performed a linkage analysis on each of these cleaned files using the multipoint Markov-chain Monte Carlo (MCMC) linkage method MCLINK [8], which computes the robust multipoint TLOD [9] statistic. For each chromosome, we identified peak regions (TLOD ≥ 2.0), not accounting for LD. To ensure that the peak could be resolved in other analyses, we included ~20 SNPs on either side of the peak and called these data our "Complete" SNP sets. These linkage results are likely to be biased because of underlying LD as 63.7% of the parents in the data set were not genotyped.

The four methods (i.e., the three novel methods and SNPLINK) were each applied to the "Complete" SNP data files. For the graphical modeling of LD combined with linkage analysis method, we used the new McLink software , which we will refer to as McLink-LD in this manuscript. For the three LD elimination methods, we used both the original MCLINK [8] and Merlin [10] software packages to perform the linkage analyses. Allele frequencies were estimated from observation of all genotyped individuals at each locus, rather than only the founders. Genotype data was available for only ~15% of the pedigree founders, and because the total number of individuals with genotype data was large (*n *= 1991 individuals) the estimated allele frequencies from all genotyped individuals provides reasonable estimates of the founder allele frequencies, even without adjustment for familial relationships. The original pedigree structure for all 757 pedigrees was used for all analyses, except for the Merlin analyses, in which 56 large pedigrees were removed because of memory limitations. For the genetic map, we assumed that 1 Mb was equivalent to 1 cM. Although this is a simplistic assumption, when inter-marker distances are relatively short and when a dense marker map is used, the assumption has been shown to produce nearly identical linkage results as a more detailed genetic map [11]. We performed a parametric analysis using a dominant model, assuming a minor allele frequency of 0.01 and a penetrance of 0.008, 0.5, and 0.5 for carriers of none, one, and two disease alleles, respectively. Our parametric model results in an overall prevalence rate of 7.5 cases per 1000 individuals, which is consistent with published prevalence rates for rheumatoid arthritis [12].

### McLink-LD

Thomas and Camp [13] and Thomas [14] introduced graphical modeling as an approach to represent allelic association in a tractable way. A graphical model consists of two elements: a Markov graph with vertices representing variables, which are connected in such a way that given the states of its neighbors, the state of a variable is conditionally independent of any other variable and the parameters that specify the conditional dependences. In the case of discrete data, these are given by multinomial distributions on the states of the variables in the maximal cliques of the graph. Thomas [14] developed a two-stage scheme to apply this to data from a random sample of diploids genotyped at multiple loci. In the first stage, an initial graphical model is assumed, and given the observed genotypes, an imputation of the haplotypes is made. In the second stage, given the imputed haplotypes a new graphical model is estimated. These stages are iterated in a simulated annealing search for an optimal LD model, and implemented in a program called HapGraph .

Through application of graphical modeling to the haplotypes of founders in a pedigree, we are able to obtain valid linkage statistics for dense SNP loci without having to discard any data. The new McLink-LD software, made available by Alun Thomas, incorporates the LD model obtained from graphical modeling and computes LOD score statistics using MCMC methods similar to those described by Thomas et al. [8] The program also models genotype errors using the approach of Thomas and Camp [15], so that checking for apparent Mendelian segregation is unnecessary. Recombination fractions can be estimated on the interval (0, 1) to take advantage of any potential evidence for linkage and to identify possible model misspecification. Full details of the method, including use of the program, are given by Thomas [16].

Although McLink-LD can estimate an LD model using a large subset of unrelated individuals and it can also model genotype errors, for consistency with our other LD elimination methods, LD assessment was performed on 100 unrelated, random individuals with genotype data selected from the 757 rheumatoid arthritis families. LD modeling using 100 individuals has been shown previously to be a sufficient sample size to capture the underlying genetic variation [17]. All SNPs in the "Complete" SNP sets were included in the analyses. Genotype errors were previously eliminated from these data files. For consistency with the other LD methods, the results displayed are over the recombination fraction interval (0, 0.5).

### PCA-haplotype method

Principal-component analysis (PCA) has been used for selection of tagging-SNPs for candidate gene studies [4,5]. Advantages of PCA include that the methodology can capture the underlying genetic variation without redundancy, genetic markers are not required to be contiguous, and statistical packages (e.g., SAS, SPSS, STATA) are readily available to perform analyses. Here we apply PCA methodology to larger genomic regions of interest using both haplotype data and genotype data. Applying the method to haplotype data, we used the same 100 unrelated, random individuals as described above as a subset of the total arthritis resource to characterize the LD structure of the regions. We performed pair-wise D' analysis for the 100 independent individuals and all pairs of SNPs in the "Complete" SNP data sets using an in-house modified version of the EMLD [18] software that increases the number of markers that can be studied at one time. All markers with a D' value ≥ 0.7 and within 2 million base pairs of each other were considered for potential removal due to high LD. These high-LD markers were phased using the software SNPHAP [19], and the resulting haplotypes were entered into PCA. Eigenvalue thresholds were set to capture at least 90% of the genetic variation of extracted factors. For each of the resulting LD groups, the SNP with the highest factor loading (required to be ≥|0.4|) was retained while all other SNPs were eliminated as providing redundant information. Linkage analysis was then performed on the "Complete" SNP set containing all 757 families but modified to only include SNPs with D' < 0.7 and SNPs selected from the PCA analysis.

### PCA-genotype method

For the PCA-Genotype method, we also used the same 100 unrelated, random individuals described above to characterize LD. For each SNP we recoded all of the genotype data from the "Complete" SNP data sets as -1, 0, 1 [20]; corresponding to homozygous wild type (1, 1), heterozygous [(1, 2) or (2, 1)], or homozygous rare genotype (2, 2), respectively. All of the recoded genotype data were then entered into the two-step PCA analysis method proposed by Horne and Camp [17]. The first PCA step was performed as described above for the PCA-Haplotype method. The second PCA step was used to select, among multiple markers in an LD group with a factor loading ≥|0.4|, the marker(s) that best capture(s) the underlying genetic variation. Because more markers are typically included in each LD group in the PCA-Genotype method compared to the PCA-Haplotype method, the two-step rather than the single-step PCA methodology was utilized here. As with the PCA-Haplotype method, we again modified the "Complete" SNP sets for all 757 families to only include markers that were retained from the PCA analysis.

### SNPLINK

SNPLINK [3] is a freely available Perl script that removes markers in high LD by computing either D' or *r*^2 ^between consecutive marker pairs for all individuals in a data set, ignoring relationships. Only one marker from each high-LD pair of SNPs, based on a high-LD threshold defined by the user, is retained. We defined high LD to be D' ≥ 0.7, consistent with our PCA-Haplotype method. We successfully analyzed the chromosome 6 "Complete" SNP data using SNPLINK, but found that SNPLINK halted unexpectedly when running the chromosome 21 data. Therefore, using the SNPLINK protocol, we manually identified which SNPs to eliminate because of high LD in both chromosomes 6 and 21. Our results for chromosome 6 compared well to the SNPLINK output. Four markers differed between the two marker lists; we selected from among two equal markers the opposite marker as that selected by SNPLINK. Hence, we feel confident that our analysis of the chromosome 21 data set would be similar to the output of SNPLINK had we been able to obtain it.

## Results and Discussion

Descriptions of the peak region including the number of markers studied and the median and interquartile distance between markers for the chromosome 6 and 21 regions are displayed in Table 1. The PCA-Genotype method resulted in the fewest number of markers studied. The PCA-Haplotype and SNPLINK methods resulted in a comparable number of markers being studied. The McLink-LD method, as described above, used the "Complete" SNP data sets.

**Table 1:** Data description across peak regions

	Chromosome 6		Chromosome 21	
		
	Number of markers	Median [interquartile] distance (Mb) between SNPs	Number of markers	Median [interquartile] distance (Mb) between SNPs
Complete SNP set	242	0.313 [0.162, 0.583]	70	0.196 [0.031, 0.443]
PCA-Haplotype	201	0.391 [0.221, 0.712]	52	0.330 [0.160, 0.569]
PCA-Genotype	79	0.833 [0.505, 2.042]	35	0.445 [0.231, 0.781]
SNPLINK	202	0.406 [0.234, 0.688]	48	0.390 [0.190, 0.582]

**Figure 1:**
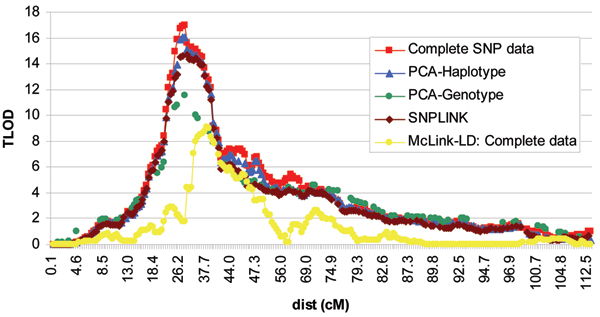
Chromosome 6 linkage results using the complete SNP set.

**Figure 2:**
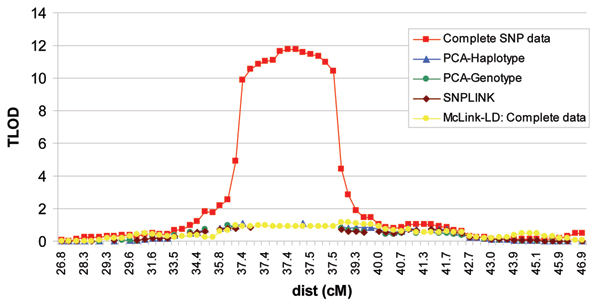
Chromosome 21 linkage results using the complete SNP set.

Graphical illustrations of the linkage results are provided for McLink-LD and the LD elimination methods analyzed in MCLINK in Figures 1 and 2. Because the Merlin peak profiles were similar to the MCLINK output, we do not provide a graphical illustration of those results. The maximum TLOD scores from MCLINK and McLink-LD and the HLOD scores from Merlin are displayed in Table 2.

**Table 2:** Maximum TLOD and HLOD scores

	Chromosome 6				Chromosome 21			
		
	MCLINK		Merlin		MCLINK		Merlin	
				
	Maximum TLOD	position	Maximum HLOD	position	Maximum TLOD	position	Maximum HLOD	position
Complete SNP set	16.95	28.96	16.40	28.96	11.75	37.44	11.31	37.46
PCA-Haplotype	16.07	28.96	15.61	28.96	1.09	37.38	1.30	37.38
PCA-Genotype	11.52	28.96	11.35	28.96	0.98	36.01	1.25	36.01
SNPLINK	14.65	28.96	14.18	33.0	0.83	37.38	0.98	36.01

	Maximum TLOD		position		Maximum TLOD		position	

McLink-LD	9.07		38.61		1.17		38.16	

The linkage results after controlling for LD all consistently verified the conclusions of Amos et al. [6], that is that the chromosome 6 peak is a true-positive result and the chromosome 21 peak is a false-positive result. For chromosome 21, all LD control methods behaved similarly with peak values of ~1.0. However, larger peak differences across methods were observed for chromosome 6 (range: 9.07 to 16.07). Peak position also varied on chromosomes 6 and 21. For chromosome 6, all methods that removed high-LD SNPs peaked at 28.96 cM, whereas McLink-LD peaked at 38.61 cM. We note that the *HLA-DRB1 *gene, the best characterized single genetic risk factor contributing to rheumatoid arthritis, maps between two SNPs included in our data sets at 28.96 cM and 33.1 cM, which is the peak location obtained from analysis of the Complete data set and all LD elimination methods. Although here we reported TLOD results as this is currently the only output of McLink-LD, rheumatoid arthritis is a complex disease, and heterogeneity LOD (HLOD) scores may more accurately reflect the true position of a peak. However, it should be noted that the HLOD scores from Merlin were reported at identical (or nearly identical) peak position values for the three LD-elimination methods using MCLINK at both the chromosome 6 and 21 regions.

While a method that incorporates the underlying LD structure should more accurately reflect the true linkage peak height and position for a chromosomal region, it may be too early to consider McLink-LD the 'gold standard'. MCMC methods can be problematic due to computational requirements and poor mixing properties and it may not be suitable for use in all cases. We note that an additional feature of the new McLink-LD program is maximization of LOD scores across recombination fractions on the (0, 1) interval. The (0, 1) interval may better be able to incorporate model errors and improve mixing properties of the graphical LD model. Maximizing over the (0, 1) interval, we observed a peak TLOD score of 13.75 at 37.04 cM, which is more similar to the results for the high-LD SNP elimination methods. However, because the high-LD SNP elimination methods all used the traditional (0, 0.5) interval, the comparison is not ideal. We also note that maximization of LOD scores over the (0, 1) interval resulted in general symmetry of the LOD function about a recombination fraction of 0.5. This is most likely due to a heavy reliance on two-generation pedigrees in the GAW15 NARAC data resource with no phase information. Any asymmetry in the chromosome 6 and 21 regions may be due to inadequate modeling of parameters [21] or failure of the sampler to mix between phase states in the few three or more generation families.

The three high-LD elimination methods all peaked at the same position, but with different peak heights. We examined residual LD between all pairs of SNPs for each of the LD elimination methods. Using a threshold for high LD of D' > 0.7, the number of pairs exceeding the threshold was less than 0.7% compared to the total number of pairwise comparisons for each method across both chromosomes, suggesting that the majority of high-LD SNPs pairs were captured. However, we do note that for chromosome 6, although the percentages were small, the number of pairs of SNPs exceeding the threshold tended to follow the same pattern as the peak TLOD results (i.e., LD threshold pattern: PCA-genotype < PCA-haplotype < SNPLINK). Thus, differences in peak height most likely are due to residual LD in each of the methods. Although not investigated here, it is also possible that the PCA-genotype method, which is the most conservative, may have diminished power compared to the other two LD elimination methods as more SNPs were eliminated. The PCA methods provided a logical approach to elimination of SNPs that were not based on a contiguous LD structure or random elimination of SNPs from a high-LD set as does SNPLINK. Because an automated PCA package for removing high-LD SNPs is yet to be developed, we recommend that PCA be used only for follow-up of regions with at least suggestive linkage evidence.

## Conclusion

Control for LD is an essential component of analyzing high-density SNP linkage data, and inflated linkage peaks may result if some method for controlling for LD is not implemented. Because we observed differences in linkage peak height and position across the four methods studied here, further work is needed to explore these and other LD control methods.

## Competing interests

The author(s) declare that they have no competing interests.
